# American Marten Respond to Seismic Lines in Northern Canada at Two Spatial Scales

**DOI:** 10.1371/journal.pone.0118720

**Published:** 2015-03-13

**Authors:** Jesse Tigner, Erin M. Bayne, Stan Boutin

**Affiliations:** Integrated Landscape Management Group, Department of Biological Sciences, University of Alberta, Edmonton, Alberta T6G 2E9, Canada; INIAV, I.P.- National Institute of Agriculture and Veterinary Research, PORTUGAL

## Abstract

Development of hydrocarbon resources across northwest Canada has spurred economic prosperity and generated concerns over impacts to biodiversity. To balance these interests, numerous jurisdictions have adopted management thresholds that allow for limited energy development but minimize undesirable impacts to wildlife. Used for exploration, seismic lines are the most abundant linear feature in the boreal forest and exist at a variety of widths and recovery states. We used American marten (*Martes americana*) as a model species to measure how line attributes influence species’ response to seismic lines, and asked whether responses to individual lines trigger population impacts. Marten response to seismic lines was strongly influenced by line width and recovery state. Compared to forest interiors, marten used open seismic lines ≥ 3 m wide less often, but used open lines ≤ 2 m wide and partially recovered lines ≥ 6 m wide similarly. Marten response to individual line types appeared to trigger population impacts. The probability of occurrence at the home range scale declined with increasing seismic line density, and the inclusion of behavioral response to line density calculations improved model fit. In our top performing model, we excluded seismic lines ≤ 2 m from our calculation of line density, and the probability of occurrence declined > 80% between home ranges with the lowest and highest line densities. Models that excluded seismic lines did not strongly explain occurrence. We show how wildlife-derived metrics can inform regulatory guidelines to increase the likelihood those guidelines meet intended management objectives. With respect to marten, not all seismic lines constitute disturbances, but avoidance of certain line types scales to population impacts. This approach provides the ecological context required to understand cause and effect relationships among socio-economic and ecological conservation goals.

## Introduction

The exploration and production of hydrocarbon resources (energy development) has increased dramatically worldwide in recent decades spurring both economic opportunities and conservation challenges. Balancing opportunities and challenges is often stymied because an understanding of the impacts to wildlife and the efficacy of mitigation options often lags behind the pace of development [1]. Across northwest boreal Canada, energy development involves the creation of linear disturbances including seismic lines, roads, and pipelines [2]. As such, a threshold-based management approach, aimed at limiting the density of linear features, has been widely suggested across the region [3, 4]. Few data exist to inform threshold calculation or density targets (but see [5]) resulting in targets ranging from 0.6 km of linear disturbance per square kilometer (km/km^2^) to 2.4 km/km^2^, in some cases calculated using all linear features ever created and in others using only certain features [6–8]. Critics contend that inconsistent density targets and a failure to account for differences in linear features confound threshold calculations and ultimately, management objectives [9].

Particularly contentious are seismic lines. Used for resource exploration, seismic lines are the narrowest, but most numerous linear disturbances associated with the energy sector in boreal Canada [3, 10]. The impact of seismic lines on wildlife, including changes to habitat use [11], home range configuration [5], and movement [12] have been clearly documented, however a detailed understanding of impacts is lacking because most studies categorize all seismic lines into a single disturbance class [11, 13, 14] (but see [15, 16]). Prior to the mid-1990s seismic lines were constructed between 6 and 10 m wide (conventional lines) [17]. In response to numerous concerns, the energy sector began constructing narrower and meandering lines collectively called low-impact seismic (LIS); currently LIS lines range in width from ≤ 2 to 5.5 m. Whether these narrower seismic lines actually mitigate impacts to wildlife is poorly understood [18] (but see [12, 15, 19]).

Until recently, the energy sector invested little effort in promoting the active recovering of vegetation along lines because seismic lines were considered temporary disturbances that would recover naturally after seismic data were acquired. However, on lines, recovery is highly variable [20]: some lines recover to heavy shrub or sapling growth over time [21, 22], but others remain in open or semi-open states for decades [17, 23, 24]. Anecdotal evidence suggests that wildlife respond to older or reclaimed seismic lines differently than open ones [25, 26], but limited research suggests the response is weak [19, 27] and poorly understood for most species.

Behavioral responses of wildlife to anthropogenic habitat disturbances (i.e., whether and how disturbances are used by individuals) play an important role in the development of management actions [28, 29]. A number of studies on wildlife response to energy development have interpreted any changes in organism behavior as inherently negative (e.g. [11, 30, 31]), and such interpretations have played a crucial role in the development of linear feature thresholds in boreal Canada. Whether statistically significant changes in behavior are indicative of biologically significant changes that impact the fitness of individuals [32], or translate into population level effects is poorly understood for most species [28, 33]. Without a clear link between behavioral and population responses, a threshold system may not achieve its intended management objectives [34], ultimately misappropriating mitigation efforts [1].

The American marten (*Martes americana*) is a medium-sized mustelid that inhabits conifer dominated forest types primarily in northern North America. Across boreal Canada marten are ubiquitous, and are a valued furbearer for many northern communities [35]. Well studied across their range, marten habitat relationships and responses to habitat disturbances triggered by forestry are well understood at multiple spatial and to a lesser degree temporal scales. At local spatial scales marten select habitat patches that provide overhead cover [36, 37] and lateral complexity [38–40]. In contrast, marten avoid naturally open stands [41, 42], and stands opened or simplified by timber harvest [36, 39, 42, 43]. Marten also use areas close to roads less than areas farther away [44]. Given sufficient recovery of overhead and structural attributes, marten will re-use regenerated habitats [45–49].

Avoidance of disturbances at fine scales can translate to changes in habitat use at broader scales, and ultimately to changes in population demographics. Compared to undisturbed landscapes, marten move longer distances [50–52] and more tortuous routes in disturbed landscapes to avoid disturbances [53]. To compensate for fine scale habitat loss, marten adopt larger home ranges in disturbed landscapes [43, 50, 54] and several studies show precipitous declines in marten use, occurrence, and occupancy of landscapes once 25%- 49% of the total area has been disturbed [39, 55–57]. Such changes are linked to lower survival [51, 52, 58, 59], reduced reproduction [59, 60], diminished body condition [52, 61] and population declines [54, 57, 59, 62] in areas with more disturbances.

To date, marten response to oil and gas development is unstudied. We measured the response of American marten to seismic lines at 2 spatial scales: the individual line and the home range. First, we measured marten response to individual seismic line types as the odds of a marten using a seismic line relative to the odds of using the forest interior. We compared use at a series of seismic line treatments that varied by width and vegetation recovery state. We expected that lines perceived by marten as disturbances would be used less frequently than forest interior, but that lines not perceived as disturbances would be used comparably to forest interior. Second, we measured marten response to seismic line density as the probability of occurrence at the home range scale across a continuum of line density. We expected that if there was a link between behavioral and population responses to seismic lines, marten avoidance of individual line types would trigger a decline in home range occurrence relative to increasing line density. Lastly, we compared the efficiency of an all-seismic line density metric to explain marten occurrence at the home range scale with other common metrics of linear feature density used in threshold calculations. We expected model fit to improve if other metrics better explained variation in marten occurrence at the home range scale.

## Materials and Methods

### Study area

**Fig 1:**
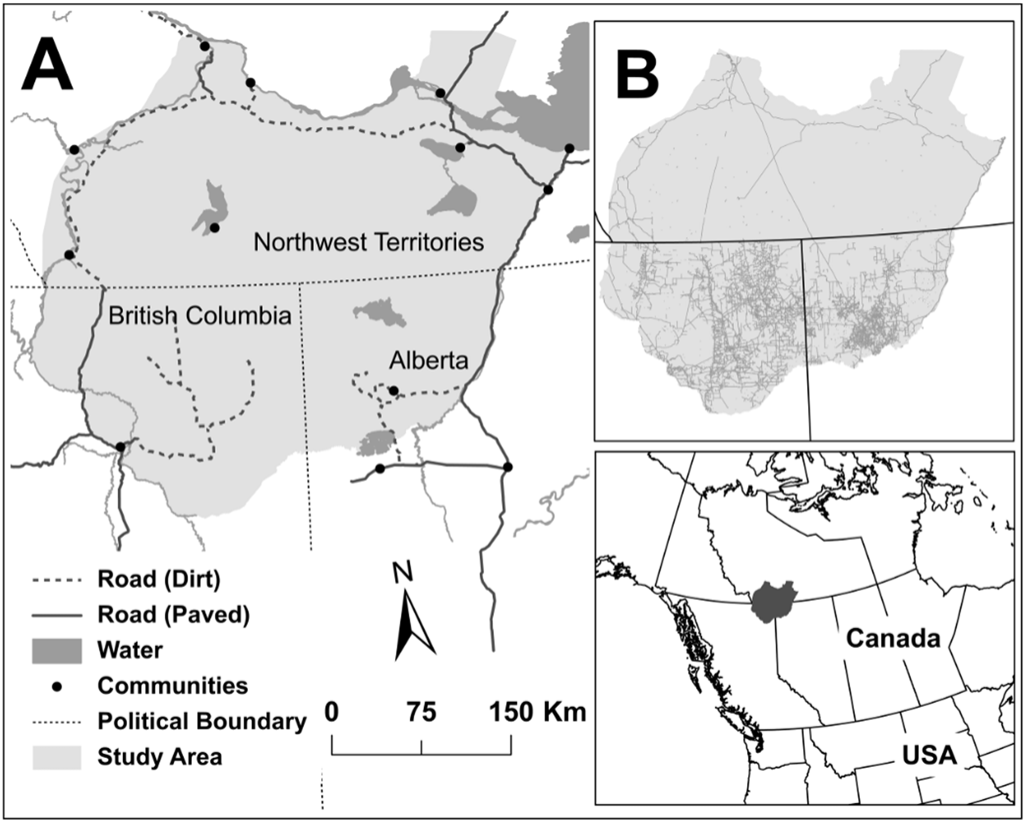
Study area. The study area in northeast Alberta, northwest British Columbia, and southwest Northwest Territories (A). Territorial and Provincial jurisdictions in western Canada are subject to divergent energy policies as evident from the extent of the disturbance footprint (shown in light grey, but excluding seismic lines for clarity) (B).

Our study area encompassed 200,000 km^2^ of northern boreal forest in northwest Alberta (AB), northeast British Columbia (BC), and southwest Northwest Territories (NWT) between 61°48′ and 58°48′ latitude and 122°41′ and 117°39′ longitude in 2008 and 2009 (Fig. 1). Energy development was the primary industrial land—use in the study area and was widespread south of the 60^th^ parallel (in AB and BC), but uncommon to the north (Fig. 1). Forestry occurs in parts of AB and BC, but we excluded these areas from our study to avoid confounding effects. Trapping for marten occurs throughout the area, but mainly where there is little or no energy development.

Forest types in this region were broadly classified into upland or lowland types [63]. Lowland forests were typified by open black spruce (*Picea mariana*) dominated muskeg and peatlands with shallow surface water, organic soils, and sparse shrub cover. By contrast, upland forests were dry and characterized by mixed stands of white spruce (*Picea glauca*) and aspen (*Populus tremuoidis and balsamifera*) (but sometimes pure conifer or deciduous) with dense shrub cover.

### Site Selection

In a Geographic Information System (GIS; ArcGIS 9.3, ESRI, Redlands, California), we reclassified Earth Observation for Sustainable Development (EOSD) [64] and Ducks Unlimited Canada Earth Cover Classification data into upland and lowland forests, and several non-forested land covers. We compiled the energy sector disturbance footprint from government, industry, and First Nations data sources and generated individual layers for seismic lines, roads, pipelines, well-pads, and oil field facilities accurate to the time of field sampling.

To isolate marten response to seismic lines and line density, we controlled for dominant forest type *a priori* using our GIS layers. Within a 5 km^2^ moving window, (average marten home range in our region [46, 65, 66]), we stratified the study area into upland and lowland forest types (≥50% of habitat within 5 km^2^), and we measured seismic line density as a continuous variable. We generated a set of 250 random candidate sites (125 each in upland and lowland forest types) across a continuum of seismic line density ranging from <0.1 km/km^2^ to >26 km/km^2^ within each forest type. Candidate sites were a minimum spacing of 5 km from each other or a human settlement, but within 15 km of road, ATV trail, or navigable water for access. In the field, if a candidate site was inaccessible or incorrectly categorized, we chose the next closest suitable site.

### Camera Trap Protocol

At each sampled site, we used a cluster of 6 remote cameras (Bushnell Scout Model 119833, Bushnell Corp. Overland Park, KS; and Reconyx RapidFire P85, Reconyx, Inc., Holmen, WI) to measure marten response to seismic line type and to seismic line density simultaneously (Government of the Northwest Territories Wildlife Research Permit No. WL-005752). All field methods were approved by the University of Alberta Animal Care and Use Committee under Protocol No. 476705. To measure marten use of seismic line type, we set up pairs of cameras with 1 on and 1 off of a seismic line (line and interior cameras, respectively). To measure marten occurrence at the home range scale we grouped 3 camera pairs together along a single seismic line to form a camera cluster. We spaced line and interior cameras by 450 m, and we spaced camera pairs within a cluster by 900 m. All cameras were left to sample for 10 trap nights (i.e. 240 hours). We considered individual cameras as the sample unit when evaluating use of seismic line types and camera clusters as the sample unit when evaluating the probability of occurrence.

We programmed all cameras to collect data 24 hours/day and to capture a photograph when a combination of motion and a change in heat (infrared energy) was detected (both camera models employed a passive-infrared sensor). We baited all cameras with 150 g of canned dog food and 50 g of tinned sardines once at camera set-up. To standardize the zone of detection for camera locations, we set baits 3–4 m from the camera at 0.5–1 m from the ground. Reported trigger speed for both camera models was < 1 second.

In 2008 we sampled using Bushnell cameras between May and September and in 2009 we used Bushnell and Reconyx cameras between May and October. Survey timing was selected to coincide with when marten are most likely to occupy established home ranges in our study area. During this time detections are more likely representative of residents’ behaviors (i.e., after mating, but before natal dispersal when marten incur longer and transient movements that may not be representative of typical habitat use or selection [52]). Camera models were not mixed within a cluster, but both models were used in upland and lowland forest types across the study area and sampled across the continuum of line density.

### Data Collection and Analysis

For our purposes use and occurrence were defined as a single photographic detection at the line and cluster level, respectively.

### Use of Seismic Line Type

**Fig 2:**
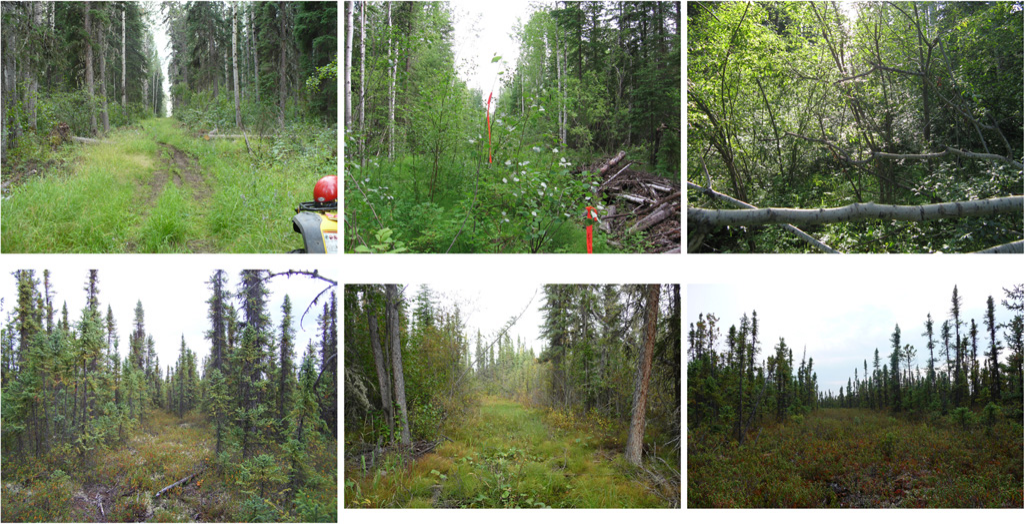
Examples of sampled seismic line types. In the field we identified and sampled along the following 6 types of seismic lines: (Top row, from upper left) 1) ≥ 6 m wide and open (open conventional); 2) ≥ 6 m wide and partially recovered (partial conventional); 3) ≥ 6 m and recovered (closed conventional); (Bottom row, from left) 4) open and ≤ 2 m wide (open 2 m); 5) open and 3–4 m wide (open 3–4 m); 6) open and 5 m wide (open 5 m). Photo credits: J. Tigner.

We evaluated marten use of seismic line types as the odds of an observation on a line relative to an observation in the forest interior (marten photographed at a camera = 1 vs. not photographed = 0). We pointed line cameras along a seismic line in 1 of the following treatments: 1) line ≥ 6 m wide and open (open conventional); 2) line ≥ 6 m wide and partially recovered (partial conventional); 3) line ≥ 6 m and recovered (closed conventional); 4) line open and ≤ 2 m wide (open 2 m); 5) line open and 3–4 m wide (open 3–4 m); 6) line open and 5 m wide (open 5 m) (Fig. 2). In the field we quantified the structural recovery state of conventional seismic lines by measuring horizontal cover (i.e., visual obstruction), shrub stem density, overhead cover, mode canopy height, coarse woody debris, and whether or not lines supported trees (woody stem ≥ 8 cm at dbh). See [20] for the full protocol description. We obtained equivalent measures in undisturbed forest plots adjacent lines for reference.

At each camera location, we classified the surrounding forest stand as upland (and noted stands as deciduous, mixed-wood, or conifer) or lowland; and we estimated stand age on a rank scale from 1 to 4, corresponding to initiation, stem exclusion, mature, and old-growth, respectively [67]. We also searched for evidence of trapping activity at camera locations, along sampled seismic lines, and within clusters.

To test for significance of seismic line use relative to the forest interior we used a population averaged generalized estimating equation (GEE) [68] with a logit link and binomial error family in Stata 11.1 IC (Stata-Corp, College Station, Texas). We used data only from those clusters where a marten was detected in this analysis to evaluate differential use of seismic line types over a 10-day exposure period. We assumed that within a cluster where marten were known to occur, detection at a camera relative to others reflected a behavioural decision [69, 70]; cameras that did not detect a marten were in areas used less than areas where a camera detected a marten. We treated camera clusters as panels and assumed an exchangeable correlation structure among cameras within each cluster. We used a semi-robust estimator of variance to generate standard errors robust to misspecification.

We assumed the likelihood of detection at a given camera remained constant over the sample period. In some cases cameras failed prior to the full 10-night sample period. We removed cameras that failed within 24 hours of set up from the analysis. For remaining cameras, we included the natural log of the total sampling duration (in minutes) per camera as an offset in our model to account for the opportunity time for each camera to capture a photograph.

We included seismic line type, forest type and age at each camera as identified in the field, occurrence of trapping, the number of other species photographed at each camera, and Julian date at camera set up in a global model. Prior to fitting the global model we tested for collinearity among variables using a threshold of |*r*| ≥ 0.7. We then removed non-significant variables using a backward stepwise procedure and retained variables at P < 0.15 in the final model, but assessed significance at P < 0.1 [71]. We also tested for an interaction between line type and cumulative seismic line density, cumulative disturbance footprint, and forest type at the cluster level obtained from the GIS to measure whether marten response to seismic line types was constant or influenced by those surrounding variables. We report results as an odds-ratio (OR) using the forest interior as the reference condition to show the direction and magnitude of difference in use between lines and the forest interior [72].

### Occurrence at the Home Range

We evaluated marten occurrence as the probability of detecting marten within a camera cluster along a continuum of seismic line density (photograph of marten in a home range = 1 vs. no photograph of marten = 0). We measured occurrence rather than occupancy because the assumption of closure is crucial to properly interpret occupancy estimates, but that assumption could not be ensured with our design [73]. Our survey duration and camera spacing were designed to garner a high likelihood of detecting marten if present [74–76] and we have no strong ecological basis with which to differentiate independent sub-surveys from our cameras into repeated surveys. We do not assume that detection probabilities are 1, and concede that the detection probability may decrease with decreasing marten density [77]. While this may underestimate an estimate of true occupancy [78], numerous studies on marten show that detection probability is high and consistent even at low densities [56, 57, 79–82], and is sufficient to directly compare occupied and non-occupied home ranges [57, 83, 84]. We assume that with our survey effort non-detections accurately reflect a reasonable estimate of true absence, and thus occurrence accurately reflects marten response to line density.

To test for significance between occurrence and line density we used the same GEE procedure and parameters described above. Here we grouped camera clusters by ecological district, a Canada-wide land cover classification (Ecological Stratification Working Group) to control for minor variations in forest structure and composition related to latitude and longitude across the study area. Clusters of mostly upland and mostly lowland forest occurred within each ecological district. We controlled for dominant forest type at the cluster scale as obtained from the GIS and for Julian date at camera set up during analysis. Significance was assessed at P < 0.1.

We then compared the relative strength of the all-seismic line density metric to explain occurrence at the home range by comparing model fit of other common metrics of linear feature density to the home range occurrence data. Specifically, we compared the all-seismic line metric (cumulative seismic density) to 1) seismic lines only, but excluding those ≤ 2 m wide (corrected seismic density); 2) roads and pipelines only (roads and pipelines); 3) roads only (roads); 4) all roads, pipelines, and seismic lines (cumulative line density); and 5) cumulative line density, but excluding seismic lines ≤ 2 m wide (corrected cumulative line density). Seismic line widths were identified from metadata attached to shape files, visually using satellite and Light Detection and Ranging (LiDAR) imagery, as measured in the field, and from discussions with energy companies active in the area. We compared the fit of each model to our data using a quasilikelihood information criterion (QIC) [85]. In all models we controlled for dominant forest type and Julian date by retaining those variables in each model run.

## Results

We deployed cameras at 1035 unique locations across 173 clusters between May and September in 2008 and May and October in 2009. Sixty-three cameras failed in < 24 hours and were removed from analyses; camera failure occurred randomly across line treatments and political jurisdiction. Twenty-two camera clusters were established differently than described in the above protocol to attain sufficient line treatment replication; these clusters were not used in occurrence analyses, but were used to group camera availability for line use analyses. We considered the remaining 151 to be home range clusters and used those for occurrence analyses. Marten were detected at 143 out of 972 (14.7%) remaining locations, and in 66 of 151 (43.7%) home range clusters (75 of 173 total clusters).

### Use of Seismic Line Type

We used data from 423 unique camera locations across 75 total clusters (open conventional: n = 39, partial conventional: n = 49, closed conventional: n = 46, open 2 m: n = 23, open 3–4 m: n = 15, open 5 m: n = 22, forest interior: n = 229). Marten response to seismic lines was significantly affected by the type of line encountered (Table 1). Line treatments with an odds-ratio > 1 were used more frequently than forest interior; those with an odds-ratio < 1 were used less frequently. Marten used open lines ≥ 3 m wide up to 90% less than forest interiors, and the odds of use of those line types relative to the odds of use of forest interiors was significant. In contrast, the odds marten used open lines ≤ 2 m wide or conventional lines supporting at least some regeneration of woody vegetation relative to the odds marten used forest interiors did not differ statistically.

**Table 1:** Behavioral response of American Marten (*Martes americana*) to seismic line types as compared to undisturbed forest interior locations in northwest Canada.

				95% Confidence Interval	
Seismic Line Type	Odds Ratio	SE	*P*	Lower	Upper
Open conventional	0.223	0.113	0.003	0.083	0.603
Partial conventional	0.819	0.253	0.519	0.448	1.500
Closed conventional	1.488	0.493	0.231	0.777	2.850
Open 2 m	0.717	0.373	0.523	0.259	1.990
Open 3–4 m	0.350	0.158	0.020	0.144	0.848
Open 5 m	0.100	0.092	0.013	0.016	0.610

An odds ratio > 1, indicates use was greater than expected relative to use of the forest interior; when < 1, use was less than expected.

Stand type at the camera was not retained in the final model indicating marten detection was not influenced by the forest type at the seismic line scale. Rank age of forest stands at the camera affected marten use (odds ratio = 1.473, *P* < 0.01); marten were more likely to use older stands (rank ages of 3 and 4) relative to younger ones (rank ages of 1 and 2). The number of other species photographed at a camera was retained in the final model (odds ratio = 0.749, *P* = 0.13) and may indicate that bait loss due to other species may influence marten finding cameras. Including or excluding any of these variables did not change the observed patterns or significance of use of seismic line types by marten. Julian date was not retained in the final model as we saw no change in marten observation with date. Evidence of trapping activity at camera locations, along sampled seismic lines, or within clusters was not retained in the final model. No tested interactions were significant indicating marten response was consistent relative to habitat type and disturbance at broader scales.

The recovery states of open, partial, and closed conventional seismic lines are shown in Table 2.

**Table 2:** Mean values (+/- **standard errors**) of vegetation attributes along conventional seismic lines at different stages of recovery (open, partial, closed) and of interior forest (interior) in northwest Canada.

		Vegetation Attributes												
		Woody Debris		Horizontal Cover^a^					Shrub density^b^	Canopy		Average DBH	Basal area	Online Trees^d^
Location	n	Count	Width	0.5m	1m	1.5m	2m	3m		Height	Closure^c^			
Open	62	**0.43** ± 0.12	**12.42** ± 1.01	**3.64** ± 0.14	**2.27** ± 0.17	**1.12** ± 0.16	**0.84** ± 0.11	**0.59** ± 0.11	**2.27** ± 0.23	**1.29** ± 0.13	**59.93** ± 3.23	n / a	n / a	**0**
Partial	73	**0.91** ± 0.14	**13.30** ± 0.93	**4.29** ± 0.08	**3.36** ± 0.11	**2.17** ± 0.17	**2.16** ± 0.13	**1.83** ± 0.13	**3.67** ± 0.20	**3.21** ± 0.19	**54.80** ± 2.61	n / a	n / a	**15**
Closed	71	**1.22** ± 0.14	**15.98** ± 1.06	**4.59** ± 0.06	**4.11** ± 0.09	**3.29** ± 0.19	**3.38** ± 0.11	**3.04** ± 0.12	**4.00** ± 0.21	**5.12** ± 0.31	**25.16** ± 2.76	n / a	n / a	**39**
Interior	206	**3.46** ± 0.24	**16.50** ± 0.85	**4.47** ± 0.04	**3.79** ± 0.06	**3.00** ± 0.18	**3.13** ± 0.07	**2.99** ± 0.07	**3.15** ± 0.15	**18.64** ± 0.55	**21.30** ± 1.65	**20.94** ± 0.71	**21.59** ± 0.91	n / a

We used mean values to categorize the recovery state of conventional lines for analyses.

^a^ Horizontal cover is a measure of visual obstruction to 1 of 6 rankings: 0, open; 1, <10% visual obstruction; 2, 10–25%; 3, 25–50%; 4, 50–75%; or 5, ≥75%. Listed rankings represent the average from 2 readings per height class (0.5 m, 1 m, 1.5 m, 2 m, and 3 m) on seismic lines (1 in each direction along the line) and 4 readings per height class in the forest interior (1 at each cardinal direction).

^b^ Shrub density is a measure of woody stems (≥0.5 m in height and <8 cm at dbh) per hectare. All stems within a 1-m × 22.6-m belt transect are tallied.

^c^ Canopy closure is a relative ranking of overhead closure where 0 is no overhead cover and 96 is 100% closure. Listed rankings represent the average of readings from 4 cardinal directions in 6 locations (i.e., 24 readings) on seismic lines and from 4 cardinal directions in 3 locations (i.e., 12 readings) in the forest interior.

^d^ Refers to the total number of seismic lines that had measureable trees (dbh ≥ 8 cm), not the average stem count per treatment. All but 1 interior plot contained measureable tree stem.

### Occurrence at the Home Range

**Fig 3:**
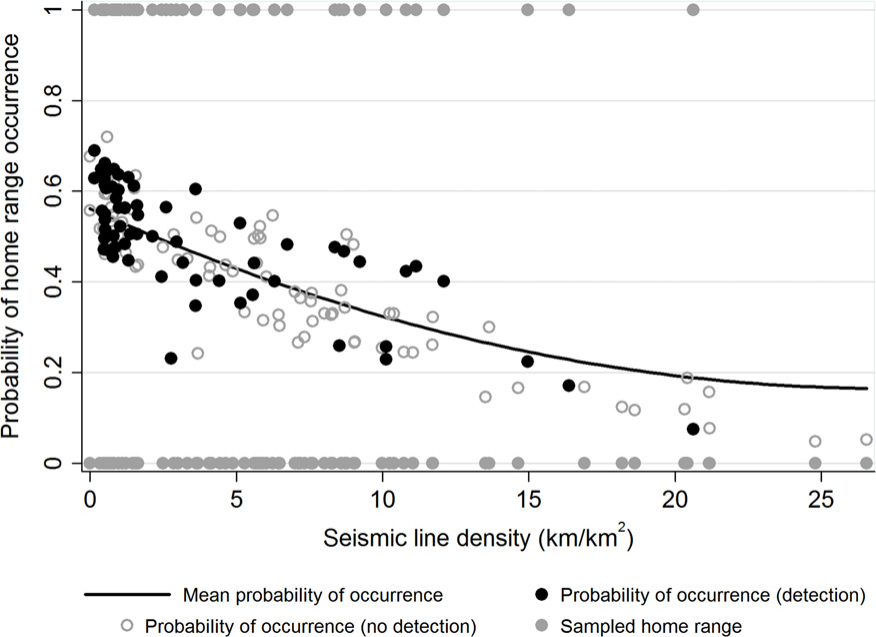
Probability of American marten occurrence relative to seismic line density. The probability that American marten (*Martes americana*) occur at the home range is significantly influenced by seismic line density in northwest Canada. As seismic line density increases, the mean probability of occurrence declines.

The probability of marten occurrence was significantly affected by seismic line density (cumulative seismic line density) at the home range scale (Fig. 3). The probability of occurrence per sampled home range ranged from a high of 0.76 in the least impacted home range (0 km/km^2^ line density) to 0.04 in the most impacted (26.4 km/km^2^). The mean probability fell from almost 60% in home ranges where seismic line density was low to approximately 20% in home ranges with the highest density of seismic lines (β = -0.083, *P* < 0.01) (Fig. 3). Interestingly, however, no marten where detected in home ranges beyond a seismic line density of 20.8 km/km^2^.

Univariate analyses showed both lowland and upland forest types affected marten occurrence at the home range scale, but that the negative effect of lowland forest was stronger than the positive effect of uplands (lowland: β = -0.487, *P* < 0.01, upland: β = 0.361, *P* < 0.01). As such, we controlled for lowland forest as the dominant forest type in our model. Dominant forest type did not have a significant effect on marten occurrence in the cumulative seismic density model, but was significant in models where seismic lines ≤ 2 m were removed from the density metrics (corrected seismic density and corrected cumulative line density). Julian date was not significant in any models.

**Fig 4:**
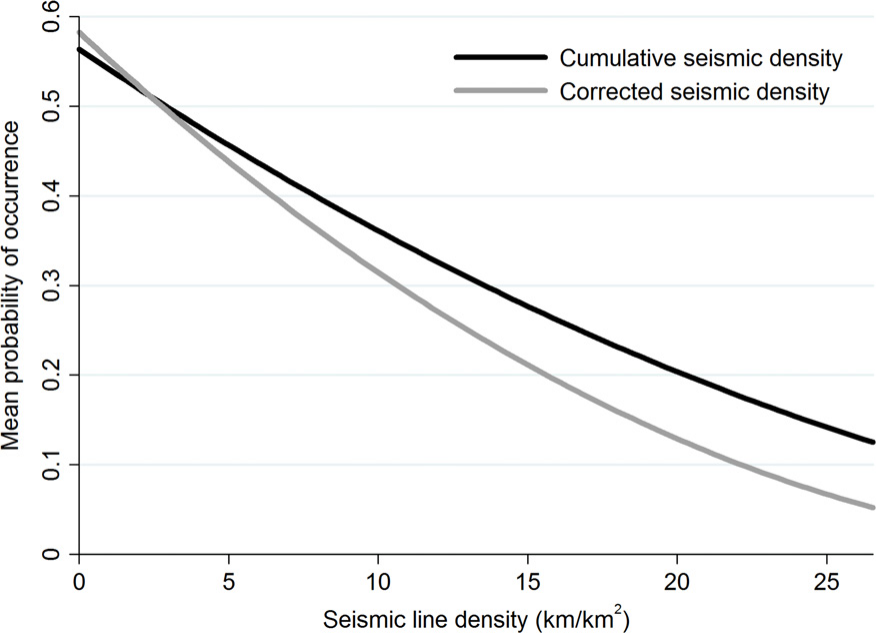
Probability of American marten occurrence is sensitive to seismic line density calculations. The relationship between the probability of American marten (*Martes americana*) occurrence and linear feature density at the home range in northwest Canada is sensitive to line density calculations. Although both metrics of seismic line density show a clear decline, where narrow seismic lines (≤ 2 m) are removed from the calculation (i.e., corrected seismic density), decline in mean probability of occurrence is almost 80%.

The metric of linear feature density that best fit our data was corrected seismic density which included all constructed seismic lines, except those ≤ 2 m wide (Table 3). However, all metrics of linear feature density that included seismic lines carry similar QIC weights suggesting all plausibly explain declining marten occurrence at the home range scale [86] (Table 3). Despite similar model weights, the shape of the relationship between line density and the mean probability of occurrence differed across models. For example, corrected seismic line density predicts a steeper and larger decline in marten occurrence than cumulative line density (Fig. 4). Corrected seismic line density estimated a decline in the mean probability of marten occurrence at the home range from almost 60% to less than 10%, representing a reduction of over 80% across the sampled continuum of seismic line density (Fig. 4).

**Table 3:** Final models comparing predicted home range occurrence of the American marten (*Martes americana*) relative to different definitions of linear feature density in northwest Canada.

Linear feature definition	β	SE	QIC	ΔQIC
**Corrected seismic density**	**-0.116**	**0.039**	**200.818**	**0.000**
Corrected cumulative line density	-0.100	0.034	202.265	1.447
Cumulative seismic density	-0.083	0.030	204.219	3.401
Cumulative line density	-0.073	0.028	205.137	4.319
Roads	0.449	0.316	212.829	12.011
Roads and pipelines	0.130	0.202	214.176	13.358

Each model controls for the proportion of lowland forest within the home range, and Julian date at survey commencement. Corrected seismic line and corrected linear feature definitions exclude seismic lines ≤ 2 m.

All metrics including seismic lines were significant in model runs (corrected seismic density: β = -0.116, *P* < 0.01; cumulative line density: β = -0.073; *P* < 0.01; corrected cumulative line density: β = -0.100, *P* < 0.01). Models that excluded seismic lines ≤ 2 m wide performed better than models that did not differentiate between seismic line types. Where seismic lines ≤ 2 m wide were removed from density metrics, average linear feature density fell from 9.436 km/km^2^ to 6.531 km/km^2^ (seismic only models), and from 10.031 km/km^2^ to 7.369 km/km^2^ (cumulative line models). The line density metrics based on roads and on roads and pipelines performed poorly and were not significant (*P* = 0.16 and *P* = 0.52, respectively).

Within group (ecological district) correlation of model residuals for the corrected seismic density model were extremely low (0.006) suggesting any spatial autocorrelation between occupied home ranges was accounted for in the model. Correlation among corrected cumulative line density residuals was similarly low (0.007). Mean probability of home range occurrence was 0.437, but that differed significantly between jurisdictions. In the NWT development is minimal and occurrence was 0.571. In BC development is widespread but employs industry best practices and occurrence was 0.436. In Alberta development is widespread, has been continuous for > 5 decades, and often uses older development methods. Occurrence in Alberta was 0.115. Relative to the NWT, occurrence was not statistically different in BC (OR = 0.754, *P* = 0.47), but was in Alberta (OR = 0.282, *P* < 0.01).

## Discussion

The goal of this study was to measure the response of American marten to seismic lines that varied by width and recovery states, to test for a link between marten response to individual lines and occurrence at a home range scale along a continuum of line density, and to compare the efficacy of common metrics of linear feature density to explain home range occurrence. Marten response to seismic lines was influenced by line width and line recovery. While marten avoided open seismic lines ≥ 3 m wide (i.e., both conventional and some types of LIS lines) compared to forest interiors, use of open lines ≤ 2 m wide and conventional lines (≥ 6 m wide) with at least partial recovery of woody vegetation was similar to use of forest interior.

Uncovering the mechanisms driving marten response to seismic lines and linear feature density was beyond the scope of this study. Still, interpreting our results within the context of marten ecology in a boreal system is useful in recommending reasonable mitigation strategies. We suspect that marten response to individual seismic lines is driven by a real or perceived risk of predation. Numerous studies show marten avoid open habitats [36, 39, 41–43], but reuse disturbed habitats with sufficient overhead and lateral cover [36, 45–49]. Marten are predated most frequently in openings [50, 87] and predated on by a variety of mammalian and avian predators [88], some of which use seismic lines as travel corridors [12, 19]. Even with high prey availability in open areas, marten do not travel far from cover [40, 56]. Despite observed avoidance of open seismic lines in this study, open linear features in the Northwest Territories support ample food resources for marten [89]. Renewed use of lines occurred with even moderate increases in woody vegetation, likely well before such increases would trigger structural shifts on lines to support forest-dwelling prey items [90–92], favored by marten [93, 94]. It is implausible trapping influenced marten use of seismic lines because we tested the response to lines only within home ranges where marten occurred. Avoidance would require marten to comprehend both a trapping risk and have some knowledge of specific trapping locations along lines.

Marten occurrence at the home range scale declined precipitously relative to increasing seismic line density. In our best model, the mean probability of occurrence declined by over 80% between the least and most impacted home ranges. This shows a clear link between a mean behavioral response of individual marten to avoid certain seismic line types and a population impact. Numerous studies show that declines in marten occurrence at a landscape scale translate to population declines [57, 60, 62], lower population density [51, 54, 77], changes in population structure [59], and reduced fitness of remaining marten [52, 61]. Other studies that link marten avoidance of fine scale disturbances to landscape abandonment typically report a threshold response (e.g. a sudden dramatic decline) beyond disturbance to 25%- 49% of study landscapes [39, 55–57]. By contrast, we observed a decline in occurrence at low levels of total habitat disturbance, and no discernable threshold response. Only ~15% of the total land base within our most heavily disturbed home range was directly impacted, and decline was consistent along the continuum of line density we sampled.

It is possible this decline is indicative of a functional habitat loss, wherein areas adjacent to, but not directly impacted by seismic lines were avoided. Functional habitat loss relative to seismic lines has also been shown in woodland caribou [11], and relative to well pads in mule deer [30, 95]. However, marten use cut block edges for travel and hunting at least proportionally to availability [53, 96] elsewhere.

Perhaps more likely is a cumulative effect of linear feature development and increased trapping pressure. Used to facilitate exploration, seismic lines are often the first or only access to remote areas and can facilitate new or increased access for trapping [4]. Where seismic exploration is promising, increased development activity ultimately results in other disturbances including well pads, roads, and pipelines. Although trapping typically wanes where extensive energy development occurs, initial access via seismic lines may have triggered over-trapping [97, 98] or trapping from which marten could not readily rebound given additional habitat disturbance from additional development activity. Regardless of whether seismic line density or trapping is the specific mechanism, we show a strong negative correlation between seismic line density and marten occurrence at the home range scale.

Several common metrics of linear feature density explained declines in home range occurrence, but others did not. QIC scores showed metrics that included seismic lines performed better than metrics that did not, and that metrics corrected for marten response to seismic line types (i.e., exclusion of seismic lines ≤ 2 m wide based on behavioral analyses) performed best [86]. This is important because we clearly show it is necessary to account for linear feature type when calculating threshold metrics. Without differentiating between a mapped and an ecologically relevant disturbance features, we actually underestimated the impact of linear feature density on marten occurrence. Compared to cumulative metrics, occurrence declined more steeply and to a lower overall probability using an accurate interpretation of line density. Of greater concern is the potential to exclude ecologically relevant disturbances from line density metrics. For example, we identified many home ranges where roads were absent or road density was low, but where seismic line density was high. If threshold metrics were based on road density alone, management would fail to prevent the significant impacts on marten related to seismic lines.

Although corrected metrics performed best, improvement was not substantial given the clear behavioral responses to seismic line types. In home ranges containing LIS lines ≤ 2 m wide, the average change between cumulative and corrected line densities was only 2.73 km/km^2^ (9.439 km/km^2^ and 6.709 km/km^2^, respectively). It is possible that line density was sufficiently high to affect occurrence regardless of how density metrics were calculated. It is also possible, that a further correction of density metrics wherein recovered lines were excluded may have improved model fit. Unfortunately, this was not possible because line recovery is poorly tied to line age [17, 20, 24, 99], nor were spatial data available for our study area to estimate current recovery states of seismic lines.

Our intention with this research was not to suggest that seismic lines are the most influential component of an energy sector disturbance footprint on marten. As discussed, seismic lines often lead to additional disturbances, all of which have been shown to impact species in different ways. Instead this work makes an important contribution to understanding the impacts of seismic lines on a species and in developing mitigation strategies based on ecologically-derived metrics.

By comparing marten use along seismic lines to use at forest interior within a population-averaged framework, we showed how individual marten would respond, on average, across a 200,000 km^2^ region to a given seismic line relative to expected use at a reference category [19, 72]. Our findings are in stark contrast to current guidelines that suggest any LIS lines are capable of mitigating behavioral impacts for wildlife, or that seismic lines constitute permanent disturbance features. Based on our study, marten make no distinction between seismic lines between 3 m and ≥ 6 m wide when open, but do distinguish open seismic lines ≥ 6 m wide as different from partially recovered and closed lines. This provides the ecological context from which to make informed management decisions on seismic lines in the boreal forest. In other words, for LIS line types to meet management expectations for marten, lines should be cut to a maximum width of ≤ 2 m wide. However, once a seismic line is cut it likely recovers to functional habitat for marten at levels well below what would be considered recovered from a forestry perspective. Clearly, the maximum line width or minimum recovery state required to mitigate the impacts of seismic lines on other species may differ [12, 15, 19]. Given a large number of wide seismic lines across boreal Canada today, additional work is needed to better understand region-specific recovery trajectories and to better identify recovery states using remote sensing technologies.

Similarly, by comparing marten occurrence at the home range along a continuum of linear feature density, we showed the expected probability of marten occurrence along that continuum [100, 101]. This provides a clear way to contextualize the ramifications of varying linear feature densities on marten in a way that can easily inform decision-making processes for setting specific thresholds. For example, in our study area, proposed regulatory thresholds for line density range from 0.6 km/km^2^ to 2.4 km/km^2^, but we observed very little change in the probability of marten occurrence between those densities. Based on our data, it seems likely that even the most liberal threshold limit in our study area is sufficient to maintain marten on a landscape subject to energy development. Whether a threshold limit of 2.4 km/km^2^ is the appropriate for northwest Canada is dependent on a myriad of socio-economic values and considerations. Regardless, we show that for marten, we can expect a 2.4 km/km^2^ threshold to incur very little cost in terms of a reduced probability of marten occurrence or a population impact relative to undisturbed landscapes. However, seismic lines are often clumped in space. Since regulatory thresholds are strongly influenced by the spatial extent at which they are applied, regulators need to be aware that portions of planning units may become unsuitable for marten if a regionally-applied threshold of 2.4 km/km^2^ is achieved without regard for the spatial patterning of seismic lines.

Lastly, we showed that a complete understanding of the cumulative impact of linear disturbances to marten at a population scale is predicated on a thorough understanding of behavioral responses to individual disturbance types. Attributing declines in species populations to a cumulative disturbance footprint based behavioral responses to individual disturbances [3, 14, 102–105] may overemphasize the impacts of disturbances thereby precluding efficient mitigation strategies. Our findings clearly show that all seismic lines do not constitute equal disturbances and that different lines may warrant different management considerations. This is mirrored in other, similarly in depth studies of species response to disturbance features that vary by attribute [32, 95, 106–108]. Too narrow a focus on mitigating the impacts of individual lines may overlook potentially substantial ecological consequences of accumulating seismic line densities at broad spatial scales.

## Supporting Information

S1 Dataset Data used to measure American marten response to seismic lines and seismic line density. (XLSX)
